# Association of Preexisting Asthma and Other Allergic Diseases With Mortality in COVID-19 Patients: A Systematic Review and Meta-Analysis

**DOI:** 10.3389/fmed.2021.670744

**Published:** 2021-06-24

**Authors:** Xianbo Wu, Yihua Xu, Lina Jin, Xiaoou Wang, Haiyan Zhu, Yiqiang Xie

**Affiliations:** ^1^School of Sports Medicine and Health, Chengdu Sport University, Chengdu, China; ^2^School of Basic Medical Sciences, Chengdu University of Traditional Chinese Medicine (TCM), Chengdu, China; ^3^Department of Respiratory, Yanbian Hospital of Traditional Chinese Medicine, Yanji City Hospital of Traditional Chinese Medicine, Jilin, China; ^4^Department of Nephrology, Yanbian Hospital of Traditional Chinese Medicine, Yanji City Hospital of Traditional Chinese Medicine, Jilin, China; ^5^Integrated Traditional Chinese Medicine (TCM) & Western Medicine Department, Clinical Medical College and The First Affiliated Hospital of Chengdu Medical College, Chengdu, China; ^6^Traditional Chinese Medicine (TCM) College, Hainan Medical University, Haikou, China

**Keywords:** COVID-19, asthma, allergy, mortality, intensive care unit, meta-analysis

## Abstract

**Background:** Respiratory viruses are known to contribute to asthma exacerbations. A meta-analysis of three studies reported no association between coronavirus disease 2019 (COVID-19) mortality and preexisting asthma. This study aimed to investigate the mortality of patients with COVID-19 in relation to preexisting asthma and other allergic diseases associated with changes in respiratory function.

**Methods:** PubMed, Embase, and the Cochrane Library were queried for papers published up to April 9, 2021: (1) population: patients who tested positive for SARS-CoV-2 according to the WHO guidelines; (2) exposure: preexisting asthma or allergic rhinitis; (3) outcomes: mortality, ICU admission, and/or hospitalization; and (4) language: English. For studies that reported adjusted models, the most adjusted model was used for this meta-analysis; otherwise, unadjusted results were used.

**Results:** Twenty-four studies (1,169,441 patients) were included in this meta-analysis. Patients who died of COVID-19 were not more likely to have preexisting asthma (OR = 0.95, 95%CI: 0.78–1.15, *P* = 0.602; I^2^ = 63.5%, P_heterogeneity_ < 0.001). Patients with COVID-19 and admitted to the ICU (OR = 1.17, 95%CI: 0.81–1.68, *P* = 0.407; I^2^ = 91.1%, P_heterogeneity_ = 0.407), or hospitalized (OR = 0.91, 95%CI: 0.76–1.10, *P* = 0.338; I^2^ = 79.1%, P_heterogeneity_ < 0.001) were not more likely to have preexisting asthma. The results for mortality and hospitalization remained non-significant when considering the adjusted and unadjusted models separately. The results from the sensitivity analyses were consistent with the primary analyses, suggesting the robustness of our results.

**Conclusion:** This meta-analysis suggests that the patients who died from COVID-19, were admitted to the ICU, or hospitalized were not more likely to have asthma.

## Background

Asthma is a chronic inflammatory condition characterized by various degrees and causes of airway inflammation (1, 2). Acute asthma exacerbations contribute significantly to the morbidity, mortality, and healthcare costs of asthma (1, 3, 4). Such exacerbations can occur in all patients in the presence of specific triggers (5).

Coronavirus disease 2019 (COVID-19) is a pandemic acute respiratory disease caused by SARS-CoV-2 (6, 7). As of November 22, 2020, the World Health Organization (WHO) reported 57.8 million cases and 1.3 million deaths, with nearly all countries being affected (8). The number of total cases of the COVID-19 continues to rise quickly, threatening thousands to millions of individuals with preexisting chronic conditions, who are disproportionately affected (9). As the number of published studies increases, there is a widening gap in knowledge due to inconsistent findings related to the influence of preexisting comorbidities on COVID-19 mortality, such as cardiovascular diseases, hypertension, diabetes, congestive heart failure, cerebrovascular disease, chronic kidney disease, chronic liver disease, cancer, chronic obstructive pulmonary disease, and HIV/AIDS (10–12).

Respiratory viruses are well-known triggers of asthma exacerbations (5, 13, 14). Besides SARS-CoV-2, SARS-CoV, and MERS-CoV, coronaviruses are respiratory viruses that are usually responsible for mild upper respiratory tract infections (about 15% of the cases of mild cold) (15) and have been implicated in asthma exacerbations (16), but the association between COVID-19 and exacerbation of asthma (including hospitalization and mortality) is unknown.

A previous meta-analysis found no association between asthma and mortality risk from COVID-19 (17), but only three studies contributed to the quantitative analysis of the impact of COVID-19 on asthma. Since this previous meta-analysis (17), additional relevant studies have been published. Therefore, this study aimed to investigate the mortality, ICU admission, and hospitalization of patients with COVID-19 in relation to preexisting asthma and other allergic diseases associated with changes in respiratory function.

## Methods

### Literature Search

This meta-analysis was conducted according to the Preferred Reporting Items for Systematic Reviews and Meta-Analyses (PRISMA) guidelines (18). The relevant articles were searched for using the PICO principle (19). PubMed, Embase, and the Cochrane Library were queried for papers published up to April 9, 2021, using the MeSH terms “COVID-19,” “Asthma,” “Rhinitis, Allergic,” “Death,” and “mortality,” as well as relevant key words. The following eligibility criteria were used: (1) population: patients who tested positive for SARS-CoV-2 according to the WHO guidelines (20); (2) no link exposure: preexisting asthma or allergic rhinitis; (3) outcomes: mortality, ICU admission, and/or hospitalization; and (4) language: English.

### Data Extraction

The study characteristics (authors, year of publication, the country where the study was performed, period, and total number, age, and sex of the patients), exposure parameters (the type of preexisting allergic disease), outcome parameters (covariates if the adjusted model were used), and primary outcome (mortality, ICU admission, and hospitalization) were extracted from the eligible papers by three authors (Haiyan Zhu, Yiqiang Xie, and Xiaoou Wang) independently. After a comparison of the extracted data, the discrepancies were resolved by discussion.

### Quality of the Evidence

The level of evidence of all articles was assessed independently by three authors (Xianbo Wu, Yihua Xu, and Lina Jin) according to the Cochrane Handbook (21) and the Newcastle-Ottawa scale (NOS) criteria (22). Discrepancies in the assessment were resolved through discussion until a consensus was reached.

### Statistical Analysis

All analyses were performed using STATA SE 14.0 (StataCorp, College Station, Texas, USA). The odds ratios (ORs) and corresponding 95% confidence intervals (CIs) were extracted and used to compare the outcomes, if available. Only the OR adjusted by the largest number of covariates was used if more than one regression model were presented. When the OR was not reported in the study, the number of events in the exposure and control groups were extracted for analysis. Statistical heterogeneity among studies was calculated using Cochran's Q test and the I^2^ index. An I^2^ index >50% and Q test *P* < 0.10 indicated high heterogeneity (21). The random-effect model was applied to all analyses. The possible publication bias was not assessed by funnel plots and Egger's test because the number of studies included in each quantitative analysis was <10, in which case the funnel plots and Egger's test could yield misleading results (21, 23).

## Results

### Selection of the Studies

**Figure 1:**
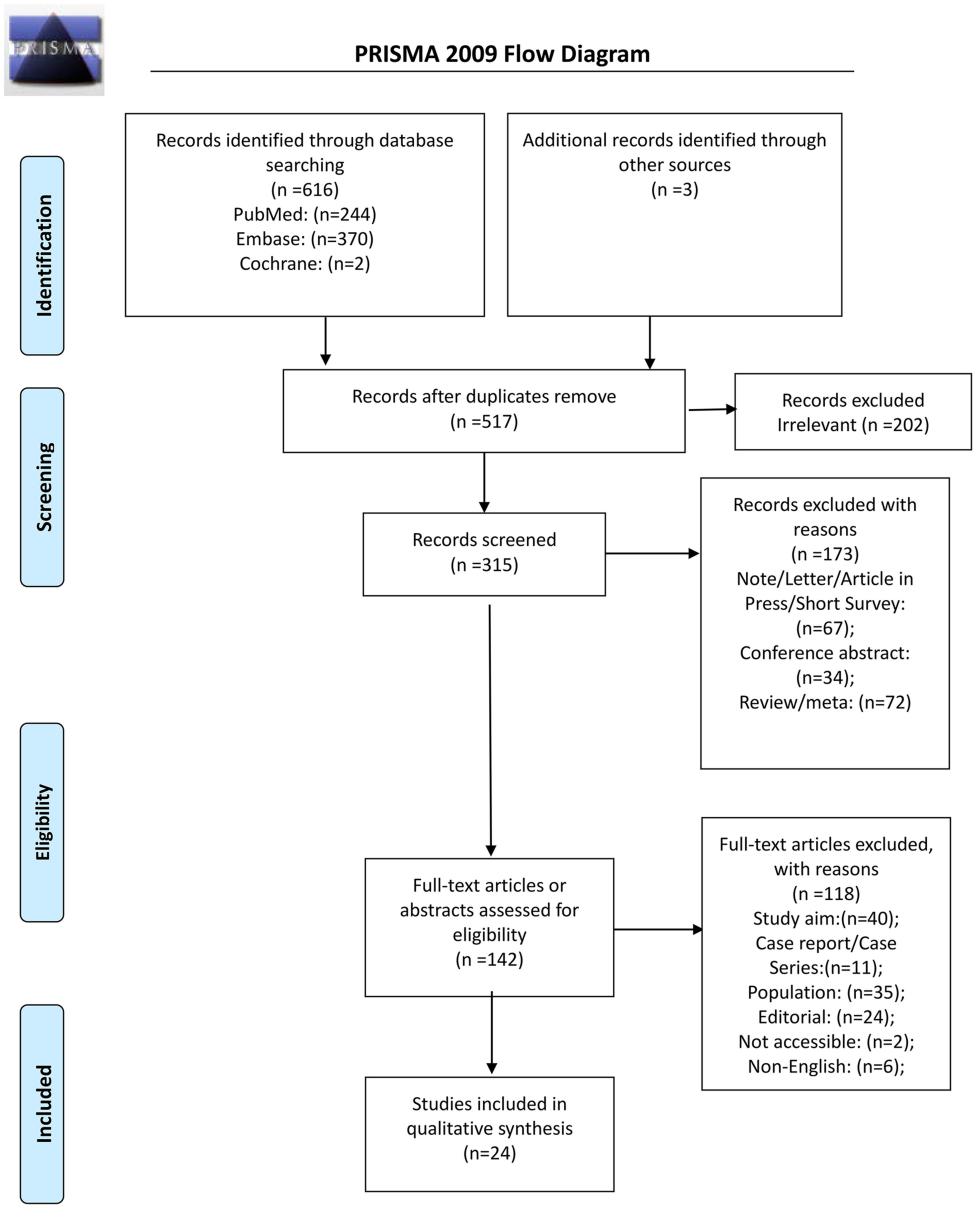
Flow diagram of study identification.

From the 616 records initially identified, 315 were screened after removing the duplicates and the irrelevant papers; 173 were excluded, and 142 full-text articles were assessed for eligibility. Among them, 118 were excluded: 40 because of study aim, 11 for study type, 35 for the population, 24 for being an editorial, two because they could be retrieved, and six non-English (Figure 1). Therefore, 24 studies were included (Supplementary Table 3).

The 24 studies included a total of 1,169,441 patients (Supplementary Table 3). Ten studies were from the United States of America, four were from the United Kingdom, two from China, one from Portugal, one from Sweden, one from Spain, one from Belgium, one from Kuwait, and three from Korea. The controls were non-asthmatic patients. Those studies were evaluated using the NOS. Three studies scored 6 points, nine scored 7 points, 11 scored 8 points, and one scored 9 points (Supplementary Table 1).

### Impact of Asthma on Mortality

**Figure 2:**
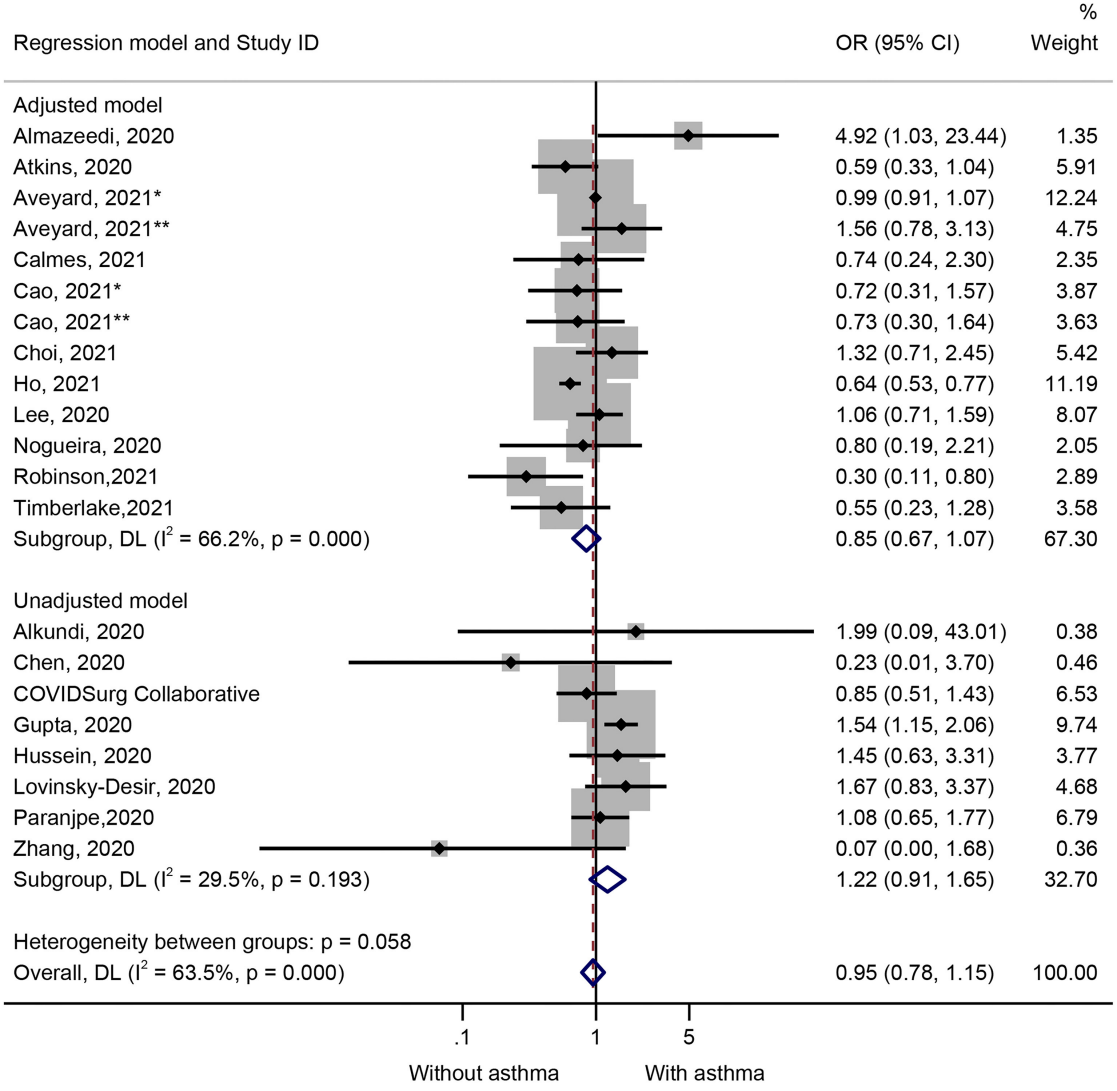
Forest plot of mortality comparing patients with or without preexisting asthma by the model applied. Weights and between-subgroup heterogeneity test are from random-effects model.

Nineteen studies (21 datasets) presented data on the impact of asthma on the mortality of COVID-19 (Figure 2). The results showed that the patients who died of COVID-19 were not more likely to have asthma (OR = 0.95, 95%CI: 0.78–1.15, *P* = 0.602; I^2^ = 63.5%, P_heterogeneity_ < 0.001) (Figure 2 and Table 1). Similar results were observed in the adjusted models (OR = 0.85, 95%CI: 0.67–1.07, *P* = 0.159; I^2^ = 66.2%, P_heterogeneity_ < 0.001) and the unadjusted models (OR = 1.22, 95%CI: 0.91–1.65, *P* = 0.190; I^2^ = 29.5%, P_heterogeneity_ = 0.193) (Figure 2 and Table 1). The sensitivity analysis showed that no single study influenced the results (Supplementary Figure 1).

**Table 1:** Subgroup analysis: treatment vs. control.

**Outcome**	**Regression model**	**No. of study**	**OR**	**95%CI**	***P***	**I-square %**	**P (Heterogeneity)**
Mortality	Overall	21	0.950	0.784–1.151	0.602	63.5	<0.001
	Adjusted model	13	0.847	0.672–1.067	0.159	66.2	<0.001
	Unadjusted model	8	1.221	0.906–1.647	0.190	29.5	0.193
ICU	Overall	15	1.167	0.810–1.681	0.407	91.1	<0.001
	Adjusted model	14	1.142	0.789–1.654	0.480	91.7	<0.001
	Unadjusted model	1	2.911	0.354–23.952	0.320	/	/
Hospitalization	Overall	10	0.914	0.761–1.098	0.338	79.1	<0.001
	Adjusted model	8	0.890	0.723–1.095	0.269	81.6	<0.001
	Unadjusted model	2	1.067	0.599–1.901	0.826	66.4	0.085

### Impact of Asthma on ICU Admission

**Figure 3:**
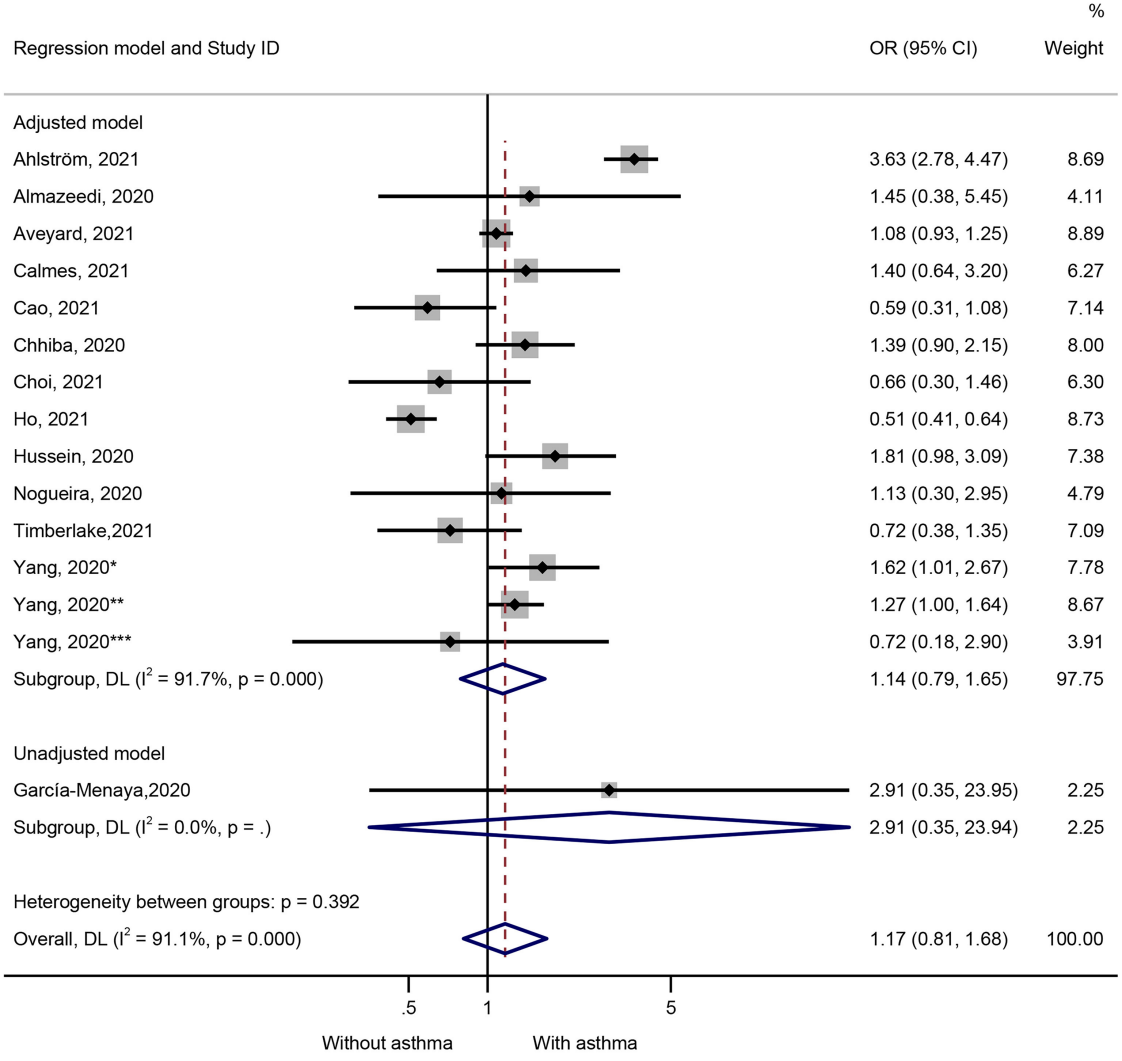
Forest plot of ICU admission comparing patients with or without preexisting asthma by the model applied. Weights and between-subgroup heterogeneity test are from random-effects model.

Fifteen studies presented data on the impact of asthma on ICU admission (Figure 3). Patients with COVID-19 and admitted to the ICU were not more likely to have preexisting asthma (OR = 1.17, 95%CI: 0.81–1.68, *P* = 0.407; I^2^ = 91.1%, P_heterogeneity_ = 0.407) (Figure 3 and Table 1). Similar results were observed in the adjusted models (OR = 1.14, 95%CI: 0.79–1.65, *P* = 0.480; I^2^ = 91.7%, P_heterogeneity_ < 0.001) and the unadjusted models (OR = 2.91, 95%CI: 0.35–23.95, *P* = 0.320) (Figure 3 and Table 1). The sensitivity analysis that no single study influenced the results (Supplementary Figure 2).

### Impact of Asthma on Hospitalization

**Figure 4:**
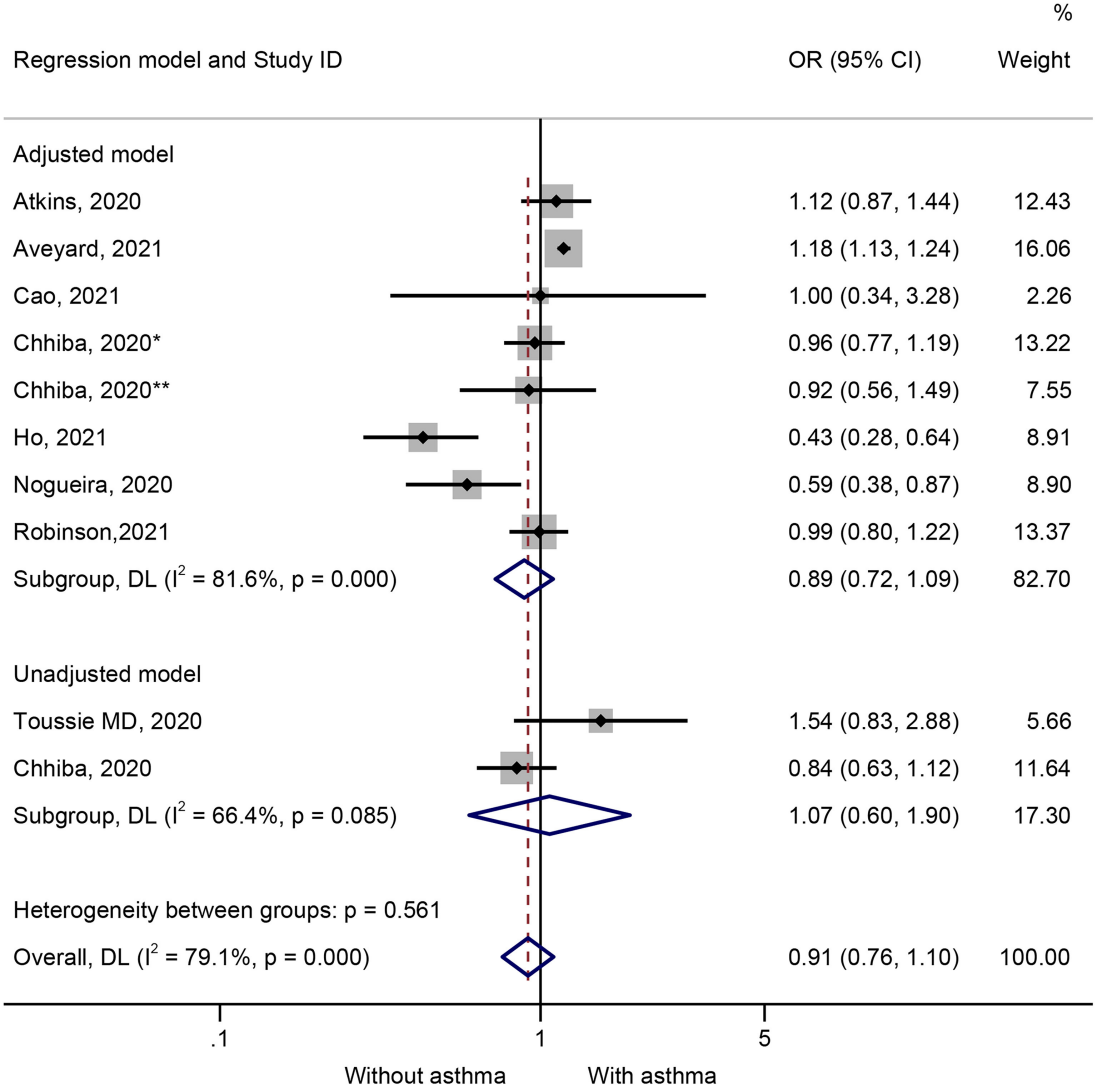
Forest plot of hospitalization comparing patients with or without preexisting asthma by the model applied. Weights and between-subgroup heterogeneity test are from random-effects model.

Ten studies presented data on the impact of asthma on hospitalization. Patients with COVID-19 and hospitalized were not more likely to have preexisting asthma (OR = 0.91, 95%CI: 0.76–1.10, *P* = 0.338; I^2^ = 79.1%, P_heterogeneity_ < 0.001) (Figure 4 and Table 1). Similar results were observed in the adjusted models (OR = 0.89, 95%CI: 0.72–1.10, *P* = 0.269; I^2^ = 81.6%, P_heterogeneity_ < 0.001) and the unadjusted models (OR = 1.07, 95%CI: 0.60–1.90, *P* = 0.826; I^2^ = 66.4%, P_heterogeneity_ = 0.085) (Figure 4 and Table 1). The sensitivity analysis that no single study influenced the results (Supplementary Figure 3).

### Publication Bias

Begg's and Egger's tests (Supplementary Table 2) and the funnel plots (Supplementary Figures 4–6) suggest no publication bias.

## Discussion

The results of this meta-analysis suggest that the patients who died of COVID-19, were admitted to the ICU, or were hospitalized were not more likely to have asthma. When available, the fully adjusted results from each study were included in the analyses, but biases from the individual studies are possible.

Despite the pandemic proportions and worldwide research focus on COVID-19, COVID-19 is a novel condition, and few studies are available on the impact of specific preexisting conditions on the outcomes of patients with COVID-19, especially asthma (24). Nevertheless, conditions like cardiovascular diseases, hypertension, stroke, diabetes, and chronic kidney diseases (6, 7, 10–12, 17, 25). The patients with such conditions are already at higher risk of morbidity and mortality because of important dysregulations of various major systems (26) that already stress and wear the regulation and adaptation abilities of the human body (27), with major impacts on the immune system. Therefore, those patients are more susceptible to major complications of COVID-19. Accordingly, previous studies showed that patients with those preexisting conditions are at higher risk of complications to seasonal influenza and previous major coronaviruses like SARS-CoV and MERS-CoV (28, 29).

Asthma is a chronic condition, and acute exacerbation of asthma is associated with significant morbidity and mortality (1, 3, 4). Some previous evidence suggested that asthma was overrepresented among hospitalized adult patients with COVID-19 (30, 31). Asthma was listed as a high-risk factor for COVID-19 because of the triggering effect of respiratory viruses on asthma exacerbations (30). Therefore, the combination of the two pulmonary conditions (asthma and COVID-19) might be associated with worse patient outcomes since respiratory viruses are known to exacerbate asthma (5, 13, 14). The meta-analysis indicates that the patients who died of COVID-19, were admitted to the ICU, or were hospitalized were not more likely to have asthma. It is supported by a smaller meta-analysis that included only three studies with data about asthma (17). Nevertheless, asthma is a heterogeneous disease characterized by different phenotypes that respond differently to treatments and have different prognoses (32–34). Future studies should examine whether specific phenotypes of asthma could be associated with worse outcomes of COVID-19. In addition, it has been suggested that asthma medications and biological agents can alleviate inflammation and enhance antivirus defenses (35, 36), but the medications were not examined in the present study.

The results of this meta-analysis must be considered in light of their limitations. As for all meta-analyses, the present study inherited the limitations of each included study, such as confounder control, selection bias, and information bias. Although the period and the inclusion criteria of each included study were similar, there were obvious differences among studies in terms of the region and age of the patients, as well as differences in the level of care they received. Those differences could explain a part of the heterogeneity observed in the results. Therefore, the random-effect model was used in all analyses to avoid overestimation. Second, the results would be more precise if all the studies had reported ORs from the adjusted model. Finally, this meta-analysis failed to examine the impact of preexisting allergies on the outcomes of COVID-19 since only two studies included patients with allergies in their analyses. More studies are still required.

## Conclusions

In conclusion, the results of this meta-analysis suggest that the patients who died from COVID-19, were admitted to the ICU, or hospitalized were not more likely to have asthma. Researchers are encouraged to design large cohort studies to investigate other preexisting risk factors.

## Data Availability Statement

The original contributions presented in the study are included in the article/Supplementary Material, further inquiries can be directed to the corresponding author/s.

## Author Contributions

XWu, HZ, and YXu conceived and coordinated the study, designed, performed and analyzed the experiments, and wrote the paper. YXi, LJ, and XWa carried out the data collection, data analysis, and revised the paper. All authors reviewed the results and approved the final version of the manuscript.

## Conflict of Interest

The authors declare that the research was conducted in the absence of any commercial or financial relationships that could be construed as a potential conflict of interest.

## Supplementary Material

Supplementary Figure 1 Sensitivity analysis of mortality.

Supplementary Figure 2 Sensitivity analysis of ICU admission.

Supplementary Figure 3 Sensitivity analysis of hospitalization.

Supplementary Figure 4 Funnel plot of mortality.

Supplementary Figure 5 Funnel plot of ICU admission.

Supplementary Figure 6 Funnel plot of hospitalization.

Supplementary Table 1 The quality assessment of included studies.

Supplementary Table 2 Publication bias of included studies.

Supplementary Table 3 Literature search and study characteristic.

## Abbreviations

COVID-19: coronavirus disease 2019
WHO: World Health Organization
PRISMA: Preferred Reporting Items for Systematic Reviews and Meta-Analyses
NOS: Newcastle-Ottawa scale
OR: odds ratio
CIs: confidence intervals.
