# Supplementary figures and images for: Association of Preexisting Asthma and Other Allergic Diseases With Mortality in COVID-19 Patients: A Systematic Review and Meta-Analysis
Source: Front Med (Lausanne). 2021 Jun 24;8:670744. doi: 10.3389/fmed.2021.670744 (PMC8264065; doi:10.3389/fmed.2021.670744)

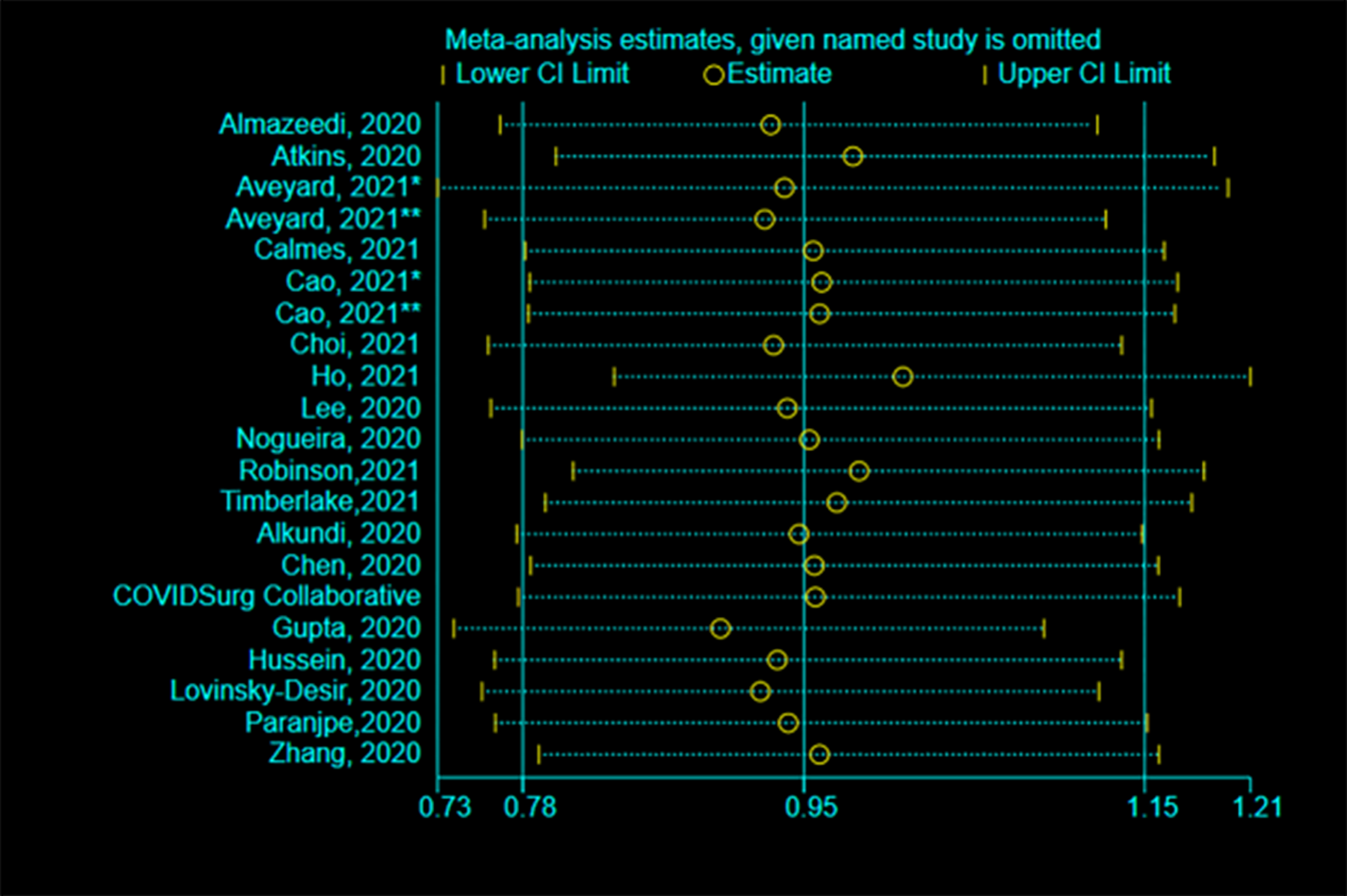

Supplement: Supplementary Figure 1 — Sensitivity analysis of mortality. [file Image_1.TIF]

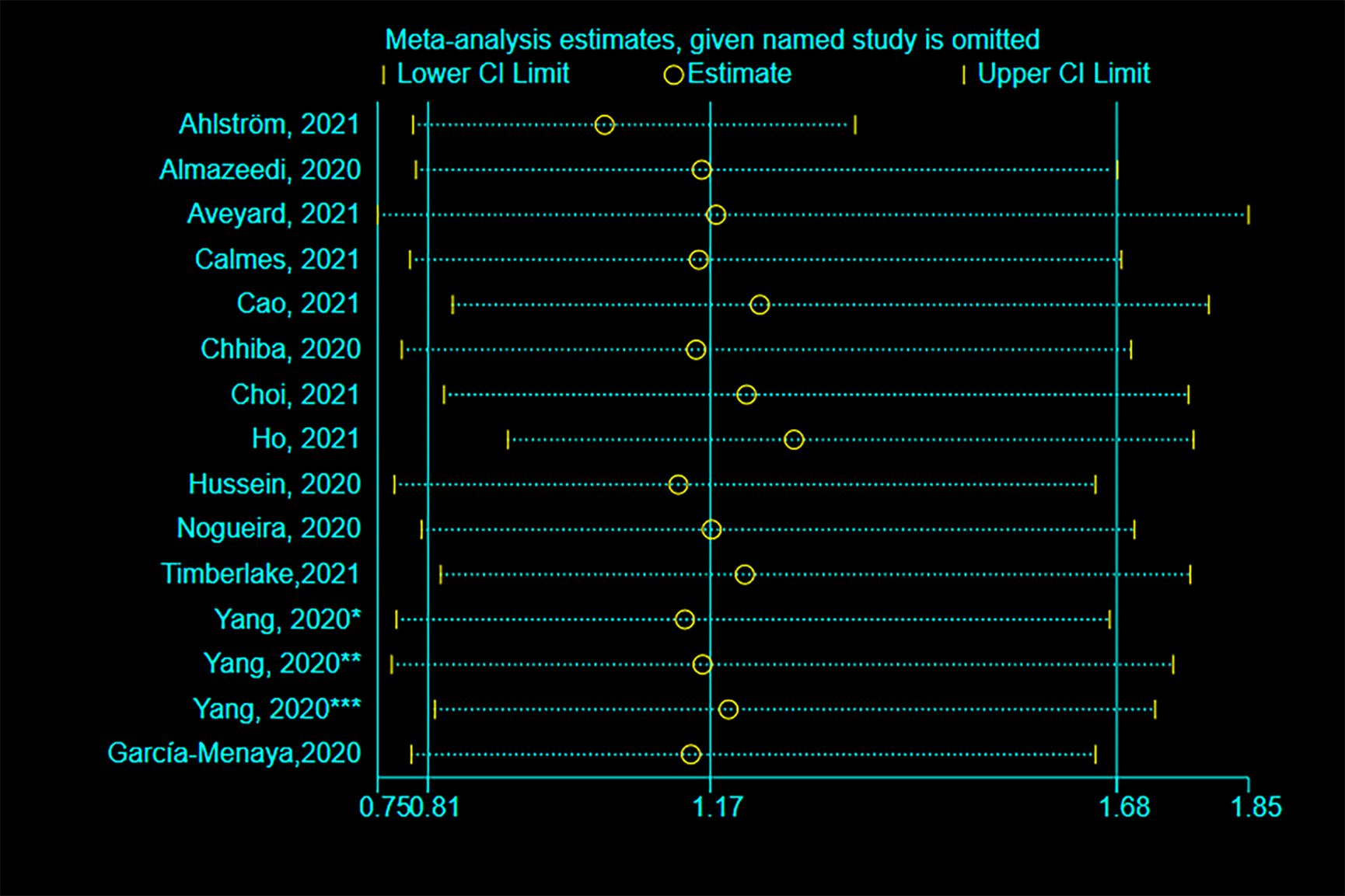

Supplement: Supplementary Figure 2 — Sensitivity analysis of ICU admission. [file Image_2.TIF]

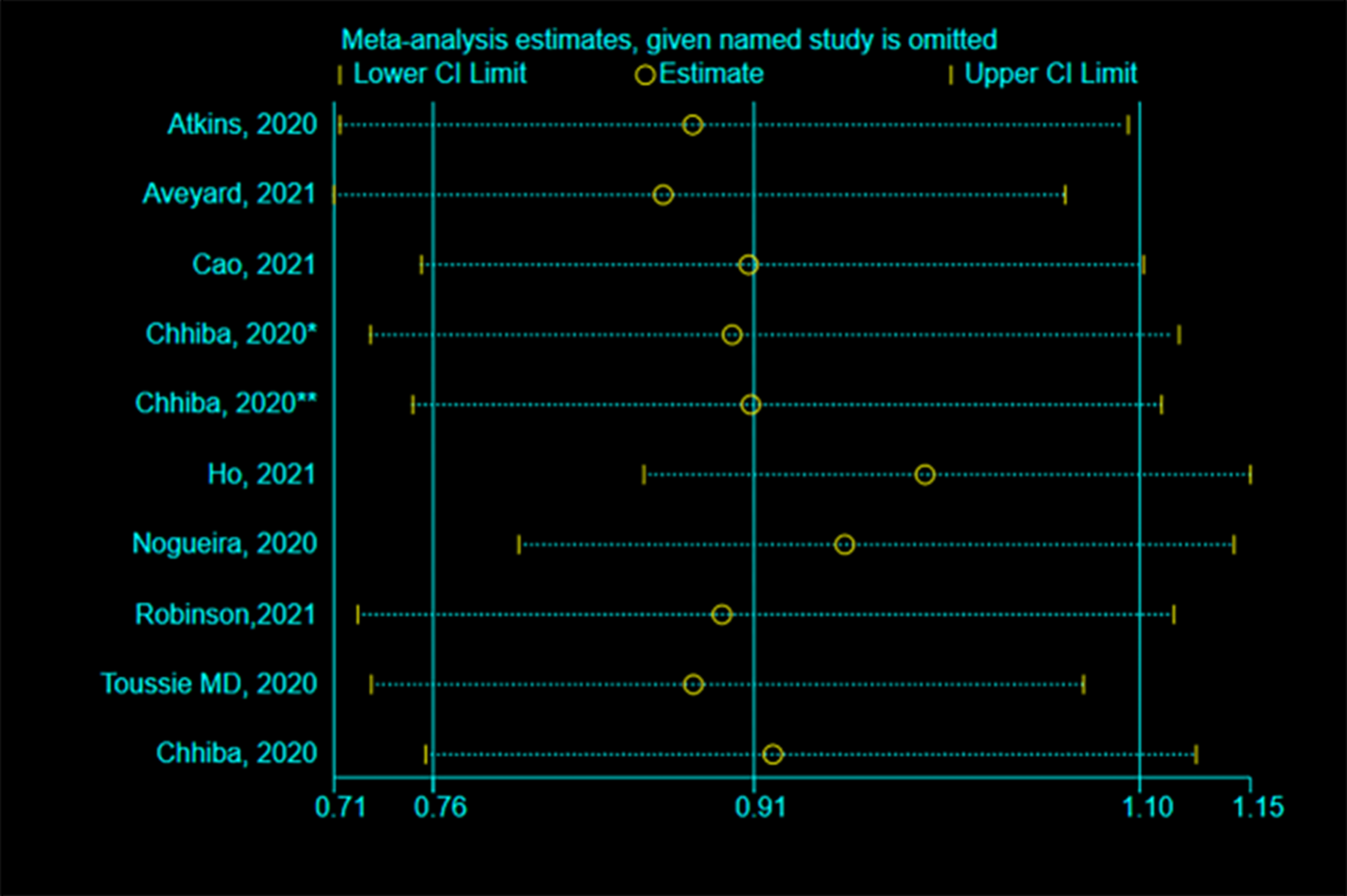

Supplement: Supplementary Figure 3 — Sensitivity analysis of hospitalization. [file Image_3.TIF]

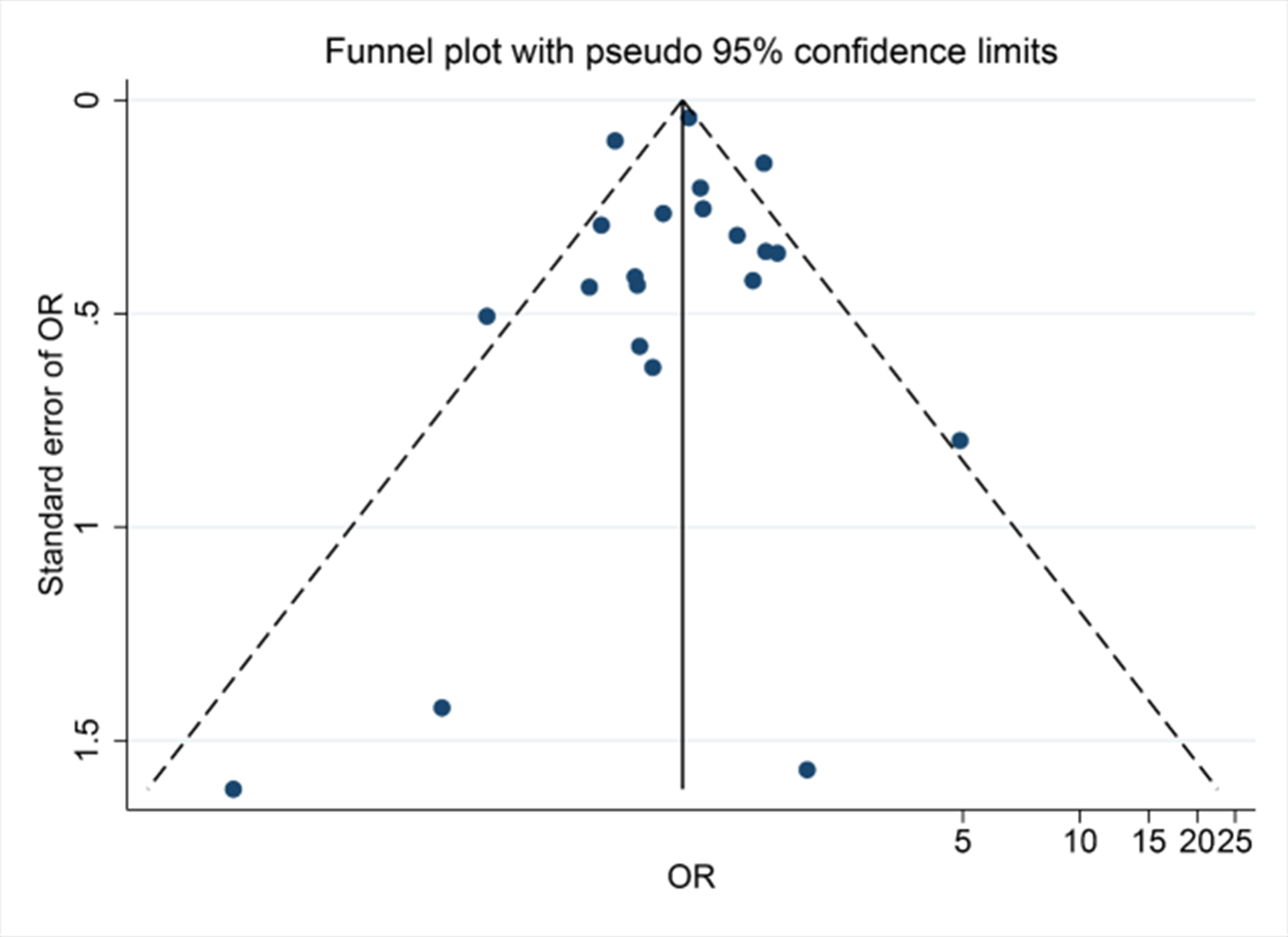

Supplement: Supplementary Figure 4 — Funnel plot of mortality. [file Image_4.TIF]

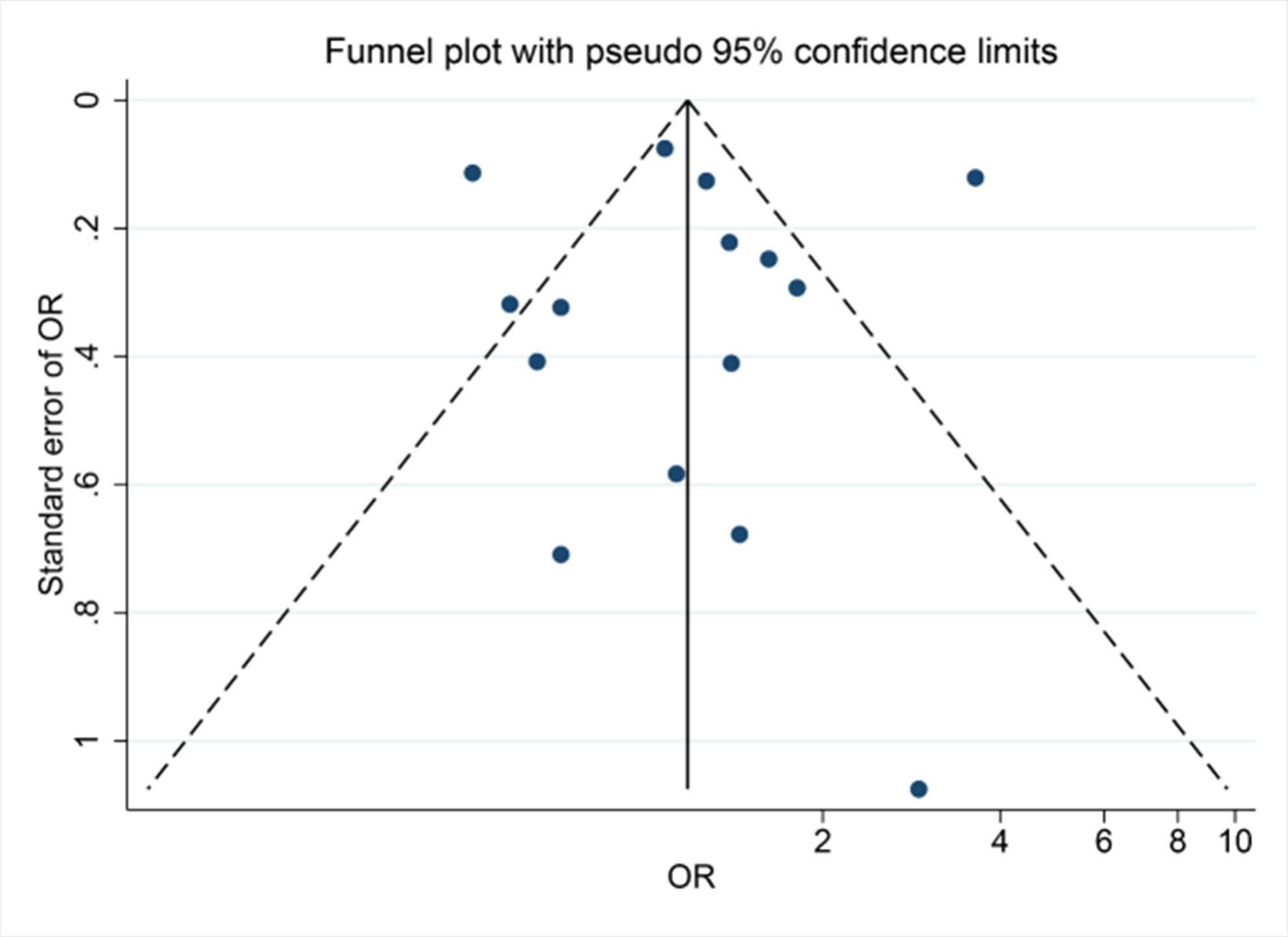

Supplement: Supplementary Figure 5 — Funnel plot of ICU admission. [file Image_5.TIF]

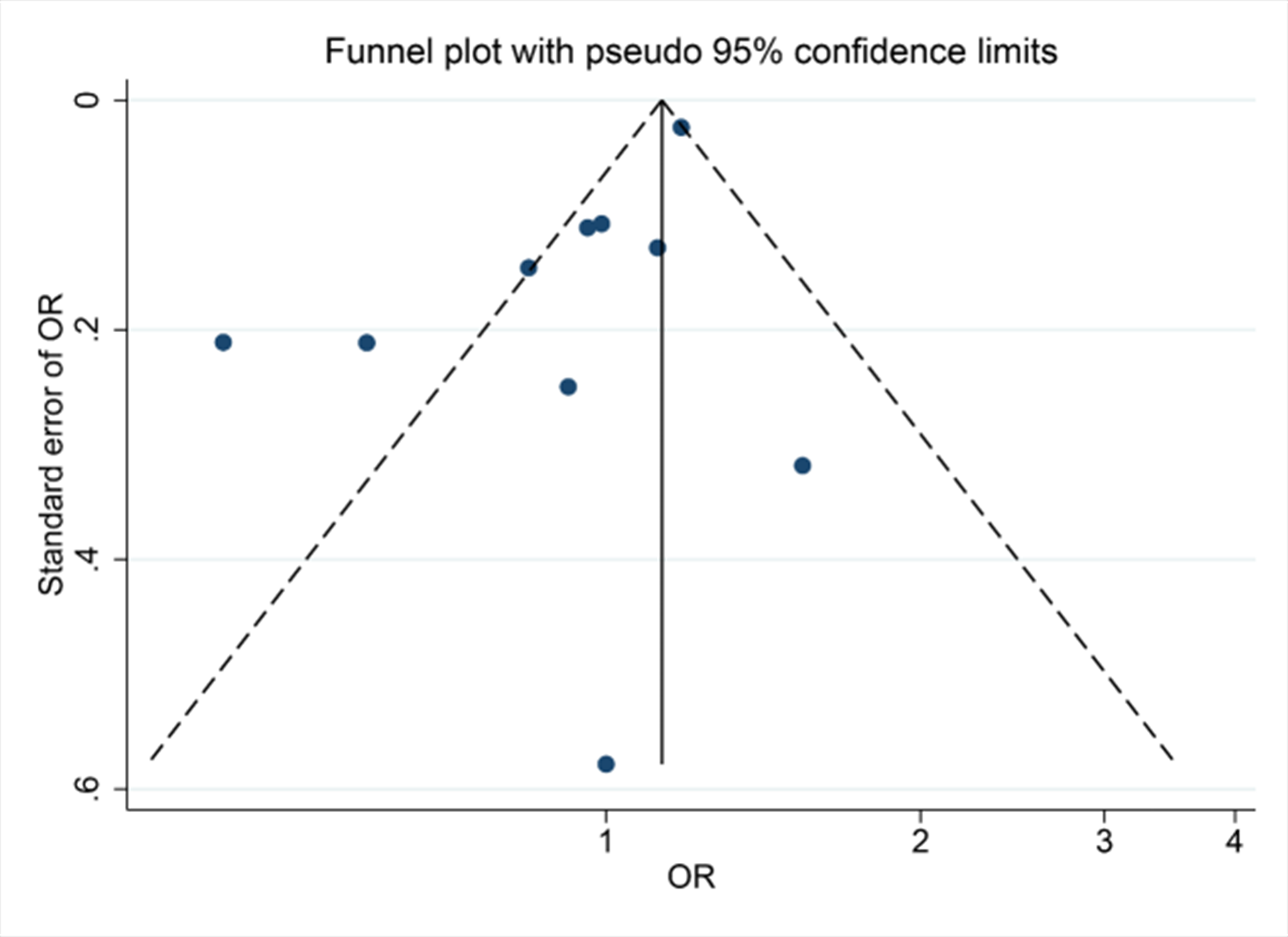

Supplement: Supplementary Figure 6 — Funnel plot of hospitalization. [file Image_6.TIF]
