# Supplementary material for: Association of Preexisting Asthma and Other Allergic Diseases With Mortality in COVID-19 Patients: A Systematic Review and Meta-Analysis
Source: Front Med (Lausanne). 2021 Jun 24;8:670744. doi: 10.3389/fmed.2021.670744 (PMC8264065; doi:10.3389/fmed.2021.670744)
Supplement: Supplementary Table 1 — The quality assessment of included studies. [file Table_1.DOCX]

**Supplementary Table S1 The quality assessment of included studies**

| **Study** | **Representativeness of the exposed cohort** | **Selection of the non-exposed cohort** | **Ascertainment of exposure** | **Demonstration that outcome of interest was not present at start of study** | **Comparability of cohorts on the basis of the design or analysis** | **Assessment of outcome** | **Was follow-up long enough for outcomes to occur** | **Adequacy of follow-up of cohorts** | **Total quality scores** |
| --- | --- | --- | --- | --- | --- | --- | --- | --- | --- |
| Ahlström, 2021 | ☆ | / | ☆ | ☆ | ☆☆ | ☆ | ☆ | ☆ | 8 |
| Alkundi, 2020 | ☆ | / | ☆ | ☆ | ☆☆ | ☆ | ☆ | / | 7 |
| Almazeedi, 2020 | ☆ | / | ☆ | ☆ | ☆☆ | ☆ | ☆ | ☆ | 8 |
| Atkins, 2020 | ☆ | / | ☆ | ☆ | ☆☆ | ☆ | ☆ | ☆ | 8 |
| Aveyard, 2021 | ☆ | / | ☆ | ☆ | ☆☆ | ☆ | ☆ | ☆ | 8 |
| Calmes, 2021 | ☆ | ☆ | ☆ | ☆ | ☆ | ☆ | ☆ | / | 7 |
| Cao, 2021 | ☆ | / | ☆ | ☆ | ☆☆ | ☆ | ☆ | / | 7 |
| Chen, 2020 | ☆ | / | ☆ | ☆ | ☆☆ | ☆ | ☆ | ☆ | 8 |
| Chhiba, 2020 | ☆ | / | ☆ | / | ☆☆ | ☆ | ☆ | / | 6 |
| Choi, 2021 | ☆ | / | ☆ | ☆ | ☆☆ | ☆ | ☆ | / | 7 |
| COVIDSurg Collaborative | ☆ | / | ☆ | ☆ | ☆☆ | ☆ | ☆ | ☆ | 8 |
| García-Menaya,2020 | ☆ | / | ☆ | ☆ | / | ☆ | ☆ | ☆ | 6 |
| Gupta, 2020 | ☆ | / | ☆ | ☆ | ☆☆ | ☆ | ☆ | ☆ | 8 |
| Ho, 2021 | ☆ | / | ☆ | ☆ | ☆☆ | ☆ | ☆ | ☆ | 8 |
| Hussein, 2020 | ☆ | / | ☆ | ☆ | ☆☆ | ☆ | ☆ | / | 7 |
| Lee, 2020 | ☆ | / | ☆ | ☆ | ☆☆ | ☆ | ☆ | ☆ | 8 |
| Lovinsky-Desir, 2020 | ☆ | / | ☆ | / | ☆☆ | ☆ | ☆ | ☆ | 7 |
| Nogueira, 2020 | ☆ | / | ☆ | ☆ | ☆☆ | ☆ | ☆ | ☆ | 8 |
| Paranjpe,2020 | ☆ | / | ☆ | ☆ | / | ☆ | ☆ | ☆ | 6 |
| Robinson,2021 | ☆ | ☆ | ☆ | ☆ | ☆☆ | ☆ | ☆ | ☆ | 9 |
| Timberlake,2021 | ☆ | / | ☆ | ☆ | ☆☆ | ☆ | ☆ | / | 7 |
| Toussie, 2020 | ☆ | / | ☆ | ☆ | ☆☆ | ☆ | ☆ | ☆ | 8 |
| Yang, 2020 | ☆ | / | ☆ | / | ☆☆ | ☆ | ☆ | ☆ | 7 |
| Zhang, 2020 | ☆ | / | ☆ | ☆ | ☆☆ | ☆ | ☆ | ☆ | 7 |
