# Supplementary material for: Association of Preexisting Asthma and Other Allergic Diseases With Mortality in COVID-19 Patients: A Systematic Review and Meta-Analysis
Source: Front Med (Lausanne). 2021 Jun 24;8:670744. doi: 10.3389/fmed.2021.670744 (PMC8264065; doi:10.3389/fmed.2021.670744)
Supplement: Supplementary Table 2 — Publication bias of included studies. [file Table_2.DOCX]

**Supplementary Table S2.** Publication bias of included studies

| Outcome | P | | No. of study needed |
| --- | --- | --- | --- |
|  | Begg's test | Egger's test |  |
| Mortality | 0.264 | 0.762 |  |
| ICU | 0.843 | 0.962 |  |
| Hospitalization | 0.858 | 0.033 | 10 |
