# Supplementary material for: Association of Preexisting Asthma and Other Allergic Diseases With Mortality in COVID-19 Patients: A Systematic Review and Meta-Analysis
Source: Front Med (Lausanne). 2021 Jun 24;8:670744. doi: 10.3389/fmed.2021.670744 (PMC8264065; doi:10.3389/fmed.2021.670744)
Supplement: Supplementary Table 3 — Literature search and study characteristic. [file Table_3.docx]

**Supplementary Table 3.** Literature search and study characteristic

| **Author,**  **Year** | **Country** | **Period** | **Adjusted for** | **N** | **Age, years case/control** | **Male, (%)  case/control** | **Race/ethnicity** | **BMI** | **Comorbidity** | **P** |
| --- | --- | --- | --- | --- | --- | --- | --- | --- | --- | --- |
|  |  |  |  |  |  |  |  |  |  |  |
| Ahlström, 2021 [1] | Sweden | 31/1/20-27/5/20 | Gender, age and SAPS3 | 1981/7924 | 61(52-69)/61(52-69) | 74/74 | / | / | / | Asthma |
| Alkundi, 2020 [2] | UK | 3/10/20-5/10/20 | Not adjusted | 87 | 70.5±15.7 | 62.5 | / | / | Diabetes | Asthma |
| Almazeedi, 2020 [3] | Kuwait | 2/24/20-4/20/20 | Age, obesity, DM, HTN, CKD, smoker, SOFA score, procalcitonin, CRP | 1096 | 41 (25-75) | 81 | Bangladesh/Egypt/India/Iran/Kuwait/Philippines/European/Other | 26.6 ± 4.6 | / | Asthma |
| Atkins, 2020 [4] | UK | 3/16/20-4/26/20 | Comorbidities, age, sex, ethnicity, and education | 507 | 74.3±4.5 | 61.3 | White/Black/South/Asian/Other (including mixed and Chinese) | / | Delirium, Pneumonia, Falls, Fragility fractures | Asthma |
| Aveyard, 2021 [5] | England | 24/1/20-30/4/20 | Age, sex, ethnicity, socioeconomic status, region of England, body-mass index, smoking status, non-smoking-related illness, smoking-related illness. | 1,090,028 | 46.65 (18.34) | 47.6 | White/Asian/Black/Chinese/Others/not recorded | BMI ≥40, 4.4% | Hypertension, Coronary heart disease, Stroke, Atrial fibrillation, Type 2 diabetes, Type 1 diabetes, Chronic kidney disease, Chronic liver disease, Chronic neurological disease | Asthma, Active asthma, Severe asthma |
| Calmes, 2021 [6] | Belgium | 18/3/20-17/4/20 | Age, gender, asthma,  COPD, and the other comorbidities with P < .10 in the models adjusted for age and gender | 103/493 | 53±18/58±19 | 44/50 | / | 28.6±7.5/27.8±5.8 | Atopy, Emphysema, Bronchiectasis, Cardiopathy, Diabetes, History of cancer, Immunosuppressive disease, Hypertension, Dyslipidemia, Obesity | Asthma, COPD |
| Cao, 2021 [7] | USA | 3/20-9/20 | Age, race (Black or not Black),  sex, chronic obstructive pulmonary disease, and obesity | 72/271 | 55.42(15.66)/62.07(15.71) | 31.9/62.6 | Black (77.8/68.9) | 36.2 (12.12)/31.03 (8.58) | Obesity, COPD, Hypertension, Diabetes | Asthma |
| Chen, 2020 [8] | China | NA-3/22/20 | Not adjusted | 548 | 56.0±14.5 | 57.1 | / | / | COPD, Diabetes, Hypertension, Coronary heart disease, Cerebrovascular disease, Hepatitis, Cancer, Renal diseases | Asthma and Other allergic diseases |
| Chhiba, 2020 [9] | USA | 3/1/20-4/15/20 | Age, sex, race, smoking, obesity, CAD, DM, HTN,  OSA, COPD, AR, rhinosinusitis, and immunodeﬁciency | 220/1306 | / | 29.1/50.1 | Non-Hispanic, African American, Non-Hispanic white, Hispanic or Latino, Non-Hispanic Asian, Other | / | / | Asthma |
| Choi, 2021 [10] | Korea | 15/5/20-1/6/20 | Age, sex, underlying disease corresponding to each category in the CCI score and/or asthma medications | 218/7372 | ≥70(27.5/11.5) | 43.6/40.7 | / | / | / | Asthma |
| COVIDSurg Collaborative [11] | UK | 1/1/20-3/31/20 | Not adjusted | 1128 | / | 511 | / | / | Current smoker, Asthma, Cancer, Chronic kidney disease, Chronic obstructive pulmonary disease, Congestive heart failure, Dementia, Diabetes, Hypertension, Myocardial infarction, Peripheral vascular disease | Asthma |
| García-Menaya, 2020 [12] | Spain | 16/3-20-24/4/20 | Not adjusted | 24/89 | 59.21 ± 16.21/69.89 ± 15.32 | 41.6/52.8 | / | / | Hypertension, heart diseases, dyslipidemia, type II diabetes, renal diseases, prothrombotic disorders, respiratory diseases, cerebrovascular diseases, cognitive impairment, psychiatric diseases, hormonal disorders, neurological diseases, digestive diseases, neoplastic diseases, morbid obesity, anaemia | Asthma or allergy |
| Gupta, 2020 [13] | USA | 3/4/20-4/4/20 | Not adjusted | 2215 | 60.5±14.5 | 64.8 | White, Black, Asian, Other, Hispanic | 30.5 (26.6-36.2) | , Hypertension, COPD, Asthma, Other pulmonary disease, Coronary artery disease, Congestive heart failure, Chronic kidney disease End-stage kidney disease, Active cancer, Immunodeficiency | Asthma |
| Ho, 2021 [14] | USA | 7/3/20-7/6/20 | Age, sex, BMI, race, COVID-19 disease severity, Charlson comorbidity index, COPD, C-reactive protein (>150 mg/L),  interleukin-6 (>80 mg/L), ferritin (>2000 ng/L), D-dimer (>2.0 mg/L), use of anticoagulation, use of corticosteroids, and smoking (current and former) | 468/10,055 | 59.38±17.75/58.31±18.86 | 31.62/55.29 | White, Black, Asian, Other, Hispanic | / | Hypertension, Diabetes, CKD, COPD, OSA, Smokers, Obese | Asthma |
| Hussein, 2020 [15] | USA | 15/3/20-9/6/20 | Age, gender, and obesity | 72/430 | 60.6 ± 13.9/60.8 ± 15.7 | 45.8/48 | African American, White, Not Reported | 35.79 ± 9.43/32.90 ± 8.32 | Hypertension, Diabetes, Chronic heart failure, Arrhythmia, COPD, Chronic kidney disease, Cancer, Coronary artery disease, Cerebrovascular disease, Obesity | Bronchial asthma |
| Lee, 2020 [16] | Korea | 20/1/20-27/3/20 | Age, sex (male), and CCI | 686/6586 | ≥ 60(43.8/27.8) | 36/40.6 | / | / | Chronic rhinitis, Hypertension, Diabetes, Dyslipidemia, IHD, Heart failure, Malignancies | Asthma |
| Lovinsky-Desir, 2020 [17] | USA | 2/11/20-5/7/20 | Not adjusted | 163/1135 | 51(27)/15(21) | 41.3 | Black, White, Asian, Other | / | / | Asthma |
| Nogueira, 2020 [18] | Portugal | 1/1/20-4/21/20 | Age, sex, pregnancy | 20,293 | 52.1±21.3 | 41.3 | / | / | Cancer, Cardiac Disease, Hematological Disorder, Diabetes, HIV/other Immune Deficiency, Kidney Disorder, Liver Disorder, Lung Disorder, Neuromuscular Disorder | Asthma |
| Paranjpe, 2020 [19] | USA | 27/2/20-2/4/20 | Not adjusted | 2199 | 65 (54–76) | 58.8 | White, Black or African- American, Asian Pacific Islander, Other, Unknown | / | Atrial fibrillation, Coronary artery disease Cancer, Chronic kidney disease, Chronic obstructive pulmonary disease, Diabetes mellitus, Heart failure Hypertension, Stroke | Asthma |
| Robinson, 2021 [20] | USA | 4/3/20-2/7/20 | Age, sex, and date of SARS-CoV-2 test date and adjusted for age, sex, race, ethnicity, payor, smoking status,  body mass index, and Charlson comorbidity index. | 562/2868 | / | 28/28 | White, Black, Asian, Other | BMI ≥40, 11%/5% | Diabetes, Hypertension, Congestive heart failure, Severe renal disease, Sickle cell disease | Asthma |
| Timberlake, 2021 [21] | USA | 3/1/20-5/5/20 | Age, sex, race, admission diagnosis (COVID-19 vs. other), COPD, CAD, and obesity | 67/208 | 57.7 (18.2)/58.0 (18.5) | 74.6/77.9 | White, Black/African- American, Other | / | Coronary artery disease, Congestive heart failure, Chronic kidney disease, COPD, Hypertension, Type 2 diabetes mellitus, Obesity | Atopic diseases |
| Toussie, 2020 [22] | USA | 3/10/20-3/26/20 | Not adjusted | 338 | 39 (31-45) | 62 | White, Asian, Black, Hispanic, Other or unknown | 31 (27–36) | Hypertension, Diabetes mellitus type II, Human immunodeficiency virus, Febrile at ED presentation | Asthma |
| Yang, 2020 [23] | Korea | 1/1/20-5/15/20 | Age, sex, region of recidence, history of DM, CAD, cerebrovascular disease, COPD, HTN, CKD, the Charlson comorbidity index, previous use of systemic glucocorticoids and immunosuppressants | 7340 | 47.1±19.0 | 40.5 | / | / | diabetes mellitus, cardiovascular disease, cerebrovascular disease, COPD, Hypertension, chronic kidney disease, | Asthma, AR, AD |
| Zhang, 2020 [24] | China | 12/29/19-2/16/20 | Not adjusted | 289 | 57 (22-88) | 154 (53.3) | / | / | Hypertension, Diabetes mellitus, Coronary heart disease, Drug hypersensitivity (self-reported), COPD, Urticaria, Others | Asthma |

DM: diabetes mellitus, SOFA: sequential organ failure assessment score, CAD: coronary artery disease, CKD: chronic kidney disease, CRP: C-reactive protein, BMI: body mass index, COPD: chronic obstructive pulmonary diseases, OSA: obstructive sleep apnea, HTN: Hypertension, AR: allergic rhinitis, CCI: Charlson comorbidity score, SAPS3: Simplified Acute Physiology Score 3

**REFERENCES**

1. Ahlstrom B, Frithiof R, Hultstrom M, Larsson IM, Strandberg G, Lipcsey M. The swedish covid-19 intensive care cohort: Risk factors of ICU admission and ICU mortality. Acta Anaesthesiol Scand. 2021. Epub 2021/01/14. doi: 10.1111/aas.13781. PubMed PMID: 33438198; PubMed Central PMCID: PMCPMC8013403.

2. Alkundi A, Mahmoud I, Musa A, Naveed S, Alshawwaf M. Clinical characteristics and outcomes of COVID-19 hospitalized patients with diabetes in the United Kingdom: A retrospective single centre study. Diabetes Res Clin Pract. 2020;165:108263. Epub 2020/06/13. doi: 10.1016/j.diabres.2020.108263. PubMed PMID: 32531325; PubMed Central PMCID: PMCPMC7283049.

3. Almazeedi S, Al-Youha S, Jamal MH, Al-Haddad M, Al-Muhaini A, Al-Ghimlas F, et al. Characteristics, risk factors and outcomes among the first consecutive 1096 patients diagnosed with COVID-19 in Kuwait. EClinicalMedicine. 2020;24:100448. Epub 2020/08/09. doi: 10.1016/j.eclinm.2020.100448. PubMed PMID: 32766546; PubMed Central PMCID: PMCPMC7335246.

4. Atkins JL, Masoli JAH, Delgado J, Pilling LC, Kuo CL, Kuchel GA, et al. Preexisting Comorbidities Predicting COVID-19 and Mortality in the UK Biobank Community Cohort. J Gerontol A Biol Sci Med Sci. 2020;75(11):2224-30. Epub 2020/07/21. doi: 10.1093/gerona/glaa183. PubMed PMID: 32687551; PubMed Central PMCID: PMCPMC7454409.

5. Aveyard P, Gao M, Lindson N, Hartmann-Boyce J, Watkinson P, Young D, et al. Association between pre-existing respiratory disease and its treatment, and severe COVID-19: a population cohort study. Lancet Respir Med. 2021. Epub 2021/04/05. doi: 10.1016/S2213-2600(21)00095-3. PubMed PMID: 33812494; PubMed Central PMCID: PMCPMC8016404.

6. Calmes D, Graff S, Maes N, Frix AN, Thys M, Bonhomme O, et al. Asthma and COPD Are Not Risk Factors for ICU Stay and Death in Case of SARS-CoV2 Infection. J Allergy Clin Immunol Pract. 2021;9(1):160-9. Epub 2020/10/11. doi: 10.1016/j.jaip.2020.09.044. PubMed PMID: 33038592; PubMed Central PMCID: PMCPMC7539890.

7. Cao L, Lee S, Krings JG, Rauseo AM, Reynolds D, Presti R, et al. Asthma in patients with suspected and diagnosed coronavirus disease 2019. Ann Allergy Asthma Immunol. 2021. Epub 2021/02/28. doi: 10.1016/j.anai.2021.02.020. PubMed PMID: 33639262; PubMed Central PMCID: PMCPMC7905379.

8. Chen R, Sang L, Jiang M, Yang Z, Jia N, Fu W, et al. Longitudinal hematologic and immunologic variations associated with the progression of COVID-19 patients in China. J Allergy Clin Immunol. 2020;146(1):89-100. Epub 2020/05/15. doi: 10.1016/j.jaci.2020.05.003. PubMed PMID: 32407836; PubMed Central PMCID: PMCPMC7212968.

9. Chhiba KD, Patel GB, Vu THT, Chen MM, Guo A, Kudlaty E, et al. Prevalence and characterization of asthma in hospitalized and nonhospitalized patients with COVID-19. J Allergy Clin Immunol. 2020;146(2):307-14 e4. Epub 2020/06/20. doi: 10.1016/j.jaci.2020.06.010. PubMed PMID: 32554082; PubMed Central PMCID: PMCPMC7295471.

10. Choi HG, Wee JH, Kim SY, Kim JH, Il Kim H, Park JY, et al. Association between asthma and clinical mortality/morbidity in COVID-19 patients using clinical epidemiologic data from Korean Disease Control and Prevention. Allergy. 2021;76(3):921-4. Epub 2020/11/30. doi: 10.1111/all.14675. PubMed PMID: 33249591; PubMed Central PMCID: PMCPMC7753771.

11. Collaborative CO. Mortality and pulmonary complications in patients undergoing surgery with perioperative SARS-CoV-2 infection: an international cohort study. Lancet. 2020;396(10243):27-38. Epub 2020/06/02. doi: 10.1016/S0140-6736(20)31182-X. PubMed PMID: 32479829; PubMed Central PMCID: PMCPMC7259900.

12. Garcia-Menaya JM, Cordobes-Duran C, Rangel-Mayoral JF, Garcia-Martin E, Agundez JAG. Outcomes and Laboratory and Clinical Findings of Asthma and Allergic Patients Admitted With Covid-19 in a Spanish University Hospital. Front Pharmacol. 2020;11:570721. Epub 2020/10/13. doi: 10.3389/fphar.2020.570721. PubMed PMID: 33041811; PubMed Central PMCID: PMCPMC7525217.

13. Gupta S, Hayek SS, Wang W, Chan L, Mathews KS, Melamed ML, et al. Factors Associated With Death in Critically Ill Patients With Coronavirus Disease 2019 in the US. JAMA Intern Med. 2020. Epub 2020/07/16. doi: 10.1001/jamainternmed.2020.3596. PubMed PMID: 32667668; PubMed Central PMCID: PMCPMC7364338.

14. Ho KS, Howell D, Rogers L, Narasimhan B, Verma H, Steiger D. The relationship between asthma, eosinophilia, and outcomes in coronavirus disease 2019 infection. Ann Allergy Asthma Immunol. 2021. Epub 2021/03/02. doi: 10.1016/j.anai.2021.02.021. PubMed PMID: 33647451; PubMed Central PMCID: PMCPMC7910126.

15. Hussein MH, Toraih EA, Attia AS, Burley N, Zhang AD, Roos J, et al. Asthma in COVID-19 patients: An extra chain fitting around the neck? Respir Med. 2020;175:106205. Epub 2020/11/21. doi: 10.1016/j.rmed.2020.106205. PubMed PMID: 33217538; PubMed Central PMCID: PMCPMC7657611.

16. Lee SC, Son KJ, Han CH, Jung JY, Park SC. Impact of comorbid asthma on severity of coronavirus disease (COVID-19). Sci Rep. 2020;10(1):21805. Epub 2020/12/15. doi: 10.1038/s41598-020-77791-8. PubMed PMID: 33311519; PubMed Central PMCID: PMCPMC7733453.

17. Lovinsky-Desir S, Deshpande DR, De A, Murray L, Stingone JA, Chan A, et al. Asthma among hospitalized patients with COVID-19 and related outcomes. J Allergy Clin Immunol. 2020;146(5):1027-34 e4. Epub 2020/08/11. doi: 10.1016/j.jaci.2020.07.026. PubMed PMID: 32771560; PubMed Central PMCID: PMCPMC7409831.

18. Nogueira PJ, de Araujo Nobre M, Costa A, Ribeiro RM, Furtado C, Bacelar Nicolau L, et al. The Role of Health Preconditions on COVID-19 Deaths in Portugal: Evidence from Surveillance Data of the First 20293 Infection Cases. J Clin Med. 2020;9(8). Epub 2020/07/30. doi: 10.3390/jcm9082368. PubMed PMID: 32722159; PubMed Central PMCID: PMCPMC7464004.

19. Paranjpe I, Russak AJ, De Freitas JK, Lala A, Miotto R, Vaid A, et al. Retrospective cohort study of clinical characteristics of 2199 hospitalised patients with COVID-19 in New York City. BMJ Open. 2020;10(11):e040736. Epub 2020/11/29. doi: 10.1136/bmjopen-2020-040736. PubMed PMID: 33247020; PubMed Central PMCID: PMCPMC7702220.

20. Robinson LB, Wang L, Fu X, Wallace ZS, Long AA, Zhang Y, et al. COVID-19 severity in asthma patients: a multi-center matched cohort study. J Asthma. 2021:1-14. Epub 2021/03/03. doi: 10.1080/02770903.2020.1857396. PubMed PMID: 33650461; PubMed Central PMCID: PMCPMC7938653.

21. Timberlake DT, Strothman K, Grayson MH. Asthma, severe acute respiratory syndrome coronavirus-2 and coronavirus disease 2019. Curr Opin Allergy Clin Immunol. 2021;21(2):182-7. Epub 2021/01/06. doi: 10.1097/ACI.0000000000000720. PubMed PMID: 33399389.

22. Toussie D, Voutsinas N, Finkelstein M, Cedillo MA, Manna S, Maron SZ, et al. Clinical and Chest Radiography Features Determine Patient Outcomes in Young and Middle-aged Adults with COVID-19. Radiology. 2020;297(1):E197-E206. Epub 2020/05/15. doi: 10.1148/radiol.2020201754. PubMed PMID: 32407255; PubMed Central PMCID: PMCPMC7507999.

23. Yang JM, Koh HY, Moon SY, Yoo IK, Ha EK, You S, et al. Allergic disorders and susceptibility to and severity of COVID-19: A nationwide cohort study. J Allergy Clin Immunol. 2020;146(4):790-8. Epub 2020/08/19. doi: 10.1016/j.jaci.2020.08.008. PubMed PMID: 32810517; PubMed Central PMCID: PMCPMC7428784.

24. Zhang JJ, Cao YY, Tan G, Dong X, Wang BC, Lin J, et al. Clinical, radiological, and laboratory characteristics and risk factors for severity and mortality of 289 hospitalized COVID-19 patients. Allergy. 2020. Epub 2020/07/15. doi: 10.1111/all.14496. PubMed PMID: 32662525; PubMed Central PMCID: PMCPMC7404752.
